# Niacin Skin Flush Backs—From the Roots of the Test to Nowadays Hope

**DOI:** 10.3390/jcm12051879

**Published:** 2023-02-27

**Authors:** Ryszard Sitarz, Dariusz Juchnowicz, Kaja Karakuła, Alicja Forma, Jacek Baj, Joanna Rog, Robert Karpiński, Anna Machrowska, Hanna Karakuła-Juchnowicz

**Affiliations:** 1 1st Department of Psychiatry, Psychotherapy and Early Intervention, Medical University of Lublin, Gluska Street 1, 20-439 Lublin, Poland; 2 Department of Psychiatry and Psychiatric Nursing, Medical University of Lublin, 20-059 Lublin, Poland; 3 Department of Forensic Medicine, Medical University of Lublin, 20-059 Lublin, Poland; 4 Department of Anatomy, Medical University of Lublin, 20-059 Lublin, Poland; 5 Department of Dietetics, Institute of Human Nutrition Sciences, Warsaw University of Life Sciences (SGGW-WULS), 02-776 Warsaw, Poland; 6 Department of Machine Design and Mechatronics, Faculty of Mechanical Engineering, Lublin University of Technology, 20-618 Lublin, Poland

**Keywords:** niacin skin flush test, niacin sensitivity, PUFA, psychosis, first episode psychosis, ultra-high risk, UHR, schizophrenia, omega-3, omega-6

## Abstract

The niacin skin flush test (NSFT) is a simple method used to assess the content of fatty acids in cell membranes and is a possible indicator of factors hidden behind various outcomes in patients. The purpose of this paper is to determine the potential usefulness of NSFT in mental disorder diagnostics along with the determination of factors that may affect its results. The authors reviewed articles from 1977 onwards, focusing on the history, variety of methodologies, influencing factors, and proposed mechanisms underlying its performance. Research indicated that NSFT could be applicable in early intervention, staging in psychiatry, and the search for new therapeutic methods and drugs based on the mechanisms of NSFT action. The NSFT can contribute to defining an individualized diet for patients and prevent the development of damaging disease effects at an early stage. There is promising evidence for supplementation with polyunsaturated fatty acids, which have a beneficial influence on the metabolic profile and are effective even in the subclinical phase of the disease. NSFT can contribute to the new classification of diseases and a better understanding of certain mental disorders’ pathophysiology. However, there is a need to establish a validated method for assessing the NSFT results.

## 1. Introduction

Focusing on the arguments of David Horrobin postulating for the evolution of human nutrition as the basis of psychotic disorders, it is necessary to realize that with a sedentary lifestyle and the Industrial Revolution, we have lost the supply of essential fatty acids in optimal amounts for the proper functioning of the nervous system [1]. According to Horrobin, a diet overloaded with saturated fats and a shortage of those essential ones in company with possibly some unfavorable genetic endowment could led to the release of psychosis from the framework of diet, strictly defined by nature for thousands of years, undeniably contributing to the development of all mankind and shaping humanity [2].

To the group of psychotic disorders belong not only schizophrenia (SCH) but also schizoaffective disorders (SCHAD) and bipolar disorder (BD), pointing to the need to recognize its physiological heterogeneity by identifying subgroups of patients sharing a common biological signature and thus enabling to conduct further research on risk, course, and treatment efficacy [3,4,5]. SCH was described by Kraepelin as “dementia praecox” in the late 19th century and renamed by Bleuler’s term “the group of schizophrenias” in the early 20th century, argued in favor of the diversified development and course of psychosis [6,7,8,9].

A growing body of scientific evidence suggests that psychosis may have a common biological basis [10,11]. Following the example of other medical fields based on etiopathophysiological premises, modern expectations focus on a personalized approach in terms of genetic, neuroscience, and behavioral sciences to better understand and reveal the contemporary taxonomy of mental illness [12]. The Bipolar–Schizophrenia Network for Intermediate Phenotypes (B-SNIP) used a biological approach to identify distinct psychosis subtypes and after examining more than 70 variables, none allowed for the classification of patients according to the Diagnostic and Statistical Manual of Mental Disorders (DSM-5) categories. Analyzes of external validators showed the advantage of a biotypic approach to identify biologically homogeneous subgroups of psychosis [13]. The literature provides genetic and biological evidence for the underlying causes of SCH and BD, including shared risk genes and overlapping family lines with psychosis diagnoses [14,15,16]. It also appears that SCHAD is used in clinical practice as an intermediate condition, with clinical manifestations of both psychosis and mood instability. This diagnosis is somewhat solid evidence of the blurring of the current classification of psychiatric disorders in the context of a diverse and arbitrary division of the image of psychosis [17].

The purpose of this paper is to update the usefulness of the NSFT with the presentation of the SKINREMS method as a simple, non-invasive, and inexpensive test for patients with symptoms of psychosis understood as SCH, BD, and SCHAD, as well as individuals at increased risk.

## 2. Materials and Methods

Literature from PubMed, Google Scholar, and Web of Science databases was extracted. It included original articles, review articles, systematic reviews, and meta-analyses published during 1977–2022. No limits were set for the publication year. The inclusion criterion was the English language. Only human studies were included in the final analysis. The literature was analyzed in terms of history, variety of methodologies, influencing factors, and proposed mechanisms of NSFT action. All SCH spectrum disorders and other psychotic disorders were chosen. The search strategy included the following keywords: (niacin skin flush test OR niacin sensitivity OR niacin) AND (psychiatry OR psychosis OR psychotic OR schizophrenia OR unipolar OR bipolar OR schizoaffective OR affective OR depression OR manic OR hypomanic OR mania OR hypomania OR Horrobin OR prostaglandin OR phospholipid OR polyunsaturated fatty acids OR PUFA OR first episode psychosis OR ultra-high risk OR UHR OR phospholipase A2 OR arachidonic acid OR linoleic acid OR alpha-linolenic acid OR omega-3 OR omega-6 OR nutrition OR diet). In addition to the literature search on databases, references included in the analyzed papers were also taken into account. Ultimately, 86 articles were estimated as relevant to the theme and included in the review.

## 3. Results

For clarity, this section has been divided into the following subsections:

### 3.1. Niacin Skin Flush Test

The NSFT is a non-invasive and repeatable test method. It involves the application of an aqueous solution of methyl nicotinate on the skin, which causes its redness. Weak response to the niacin skin flush test (NSFT) related to SCH is widely known and observed in repeated studies, differing in methodology [18,19,20,21]. These include those where niacin was taken orally and the temperature changes were measured in the core body or on the earlobe [22,23]. Consistent with the current state of knowledge that SCH is a heterogeneous mental disease in terms of pathophysiology, the reaction to the NSFT turns out to be etiologically diverse [3,24]. Moreover, it seems that attenuated or delayed skin flushing does not always occur in patients with SCH, because even strong reactions to niacin have been reported [25]. This inconsistency in observations may be explained by the surprising results of a study by Berger et. al, which proved a significantly increased reaction in ultra-high risk (UHR) patients. Such sensitivity was inversely correlated with omega-3 and -6 fatty acids levels, but positively with phospholipase A2 (PLA2). It was concluded that the emergence of psychosis could be reflected in a “pro-inflammatory state” [26].

Tavares et al. showed in their study a significantly higher PLA2 activity among individuals with SCH [27]. Thus, the cascade of prostaglandin-forming reactions seems to be the key to the test. Furthermore, such excessive activation leads to the intensified synthesis of cyclooxygenases causing inflammatory reactions [28]. If the disturbances are similarly present in the brain area, they can significantly affect its functions influencing regional cerebral blood flow along with the neuronal activity [29]. Adding these observations to the hypothesis of neurodevelopment disturbance affecting both autonomic and higher cortical functions, Nilsson et al. showed poor test results of niacin non-responders in the cognitive aspect. In a study, as might be expected, non-responders showed not only lower IQ scores but also impaired psychomotor functions [30].

#### 3.1.1. Pathophysiological Background of the NSFT

**Figure 1:**
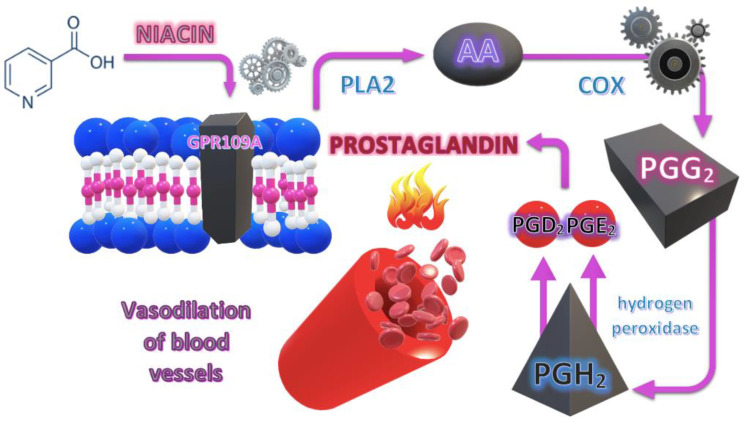
Mechanism of the niacin action on the cell membrane. PLA2—phospholipase A2, AA—arachidonic acid, COX—cyclooxygenase, PGG_2_—prostaglandin G_2_, PGH_2_—prostaglandin H_2_, PGD_2_—prostaglandin D_2_, PGE_2_—prostaglandin E_2_.

The process is significantly influenced by the hydroxycarboxylic acid receptor 2, which is coupled with the G protein and is expressed on the immune cells of the epidermis and keratinocytes. Interaction between niacin and niacin G protein-coupled receptor HM74A on epidermal cells increases the concentration of cytosolic calcium which triggers phospholipase A2 activity [31]. Thus, the hydrolysis of membrane phospholipids occurs as well as the release of AA, which is converted to prostaglandin D2 (PGD2) and prostaglandin E2 (PGE2) via cyclooxygenase-2 (COX-2). This reaction leads to the relaxation of the smooth muscle, causing the dilation of blood vessels [32,33]. Therefore, it has been postulated that the decreased skin reaction in patients with SCH may be caused by AA deficiency in the cell membrane [34,35,36]. The mechanism of the niacin action on the cell membrane is presented in Figure 1. 

#### 3.1.2. Measurement Methods Used in NSFT

Studies using NSFT differ in terms of methodology. So far, the methods used could be divided into those based on (1) thermal, (2) optical, and (3) blood flow and optical spectroscopy change measurements.

In 1980, David Horrobin maintained that patients suffering from schizophrenia need more than 250 mg of niacin, taken orally, than normal individuals to flush [37]. In the 1990s, Rybakowski et al. conducted a study by administering 200 mg of oral niacin followed by thermometric recordings. Flushing was observed at the face, neck, and chest levels. Using an electronic thermometer, the temperature of the left earlobe was measured [23]. In addition, the body temperature measurement method was used by Glen et al. where, after oral intake of 200 mg of nicotinic acid, body temperature was measured using an oral thermometer and skin temperature by electrodes placed on both earlobes. Niacin response was defined as an increase in skin temperature of 2 degrees Celsius or more [34].

A very common method of conducting NSFT research is the optical one. It assumes observing the skin reaction at various time intervals and then documenting local redness, most often in the area of the forearm. Predominantly three or four concentrations of aqueous methyl nicotinate (i.e., 0.1 M, 0.01 M, 0.001 M, and 0.0001 M) are used. By tissue papers of the same size, methyl nicotinate is applied to the skin for a certain time. In this methodology, a topical skin reaction is assessed subjectively by flush status, often based on measurement scales created especially for these purposes, i.e., 0 = no reaction, 1 = minimal redness, 2 = moderate redness, and 3 = maximal redness. In addition, edema associated with local redness is sometimes described [18,27,38,39].

Since significant cutaneous vasodilation can occur in the absence of visually detectable edema, some studies measure skin changes using Doppler flowmetry [20,40,41]. The use of specialized equipment is also associated with the measurement of skin color changes using optical reflection spectroscopy [42].

##### SKINREMS—An Innovative Measurement Device for NSFT Assessment

The authors would like to present an alternative measurement method—SKINREMS as an innovative device for NSFT assessment [33].

The NSFT method assumes the application of methyl nicotinate solutions (Sigma Chemical, St. Louis, MO, USA) in three concentrations (0.1 M, 0.01 M, and 0.001 M) sprinkled on 2 × 2-cm tissue paper to the forearm skin for 90 s. The subsequent examination period lasts 15 min when the patient holds their forearm in the designed measuring device.

**Figure 2:**
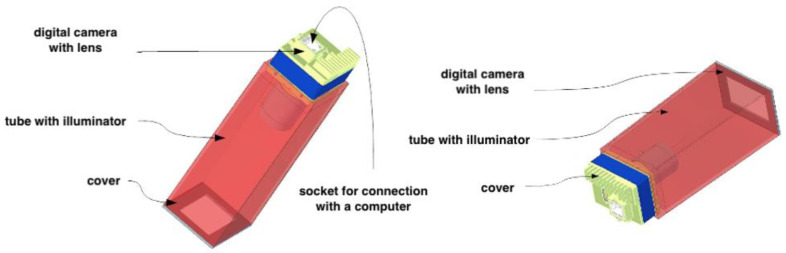
The scheme shows the first draft of the SKINREMS device, created by an interdisciplinary team of researchers [33].

A multidisciplinary team of researchers from the chair of the Department of Psychiatry, Psychotherapy and Early Intervention, Medical University of Lublin, designed and developed a novel measurement system for NSFT assessment. The initial design of the device is presented in Figure 2, which is distinguished by the simplicity of construction and low economic outlay. Thanks to the ergonomic design, patient examination is possible in all conditions.

The measuring device consists of a tightly closed tube, which is evenly illuminated inside and has an entrance for a camera lens, which during the examination registers skin changes based on a fifteen-minute time-lapse movie, in which the photo is captured every 30 s, and thus the examination undergo dynamic changes. A total of 90 images are obtained per patient for all three concentrations of methyl nicotinate. The observed formation, disappearance, and intensity of the reaction differ over time.

**Figure 3:**
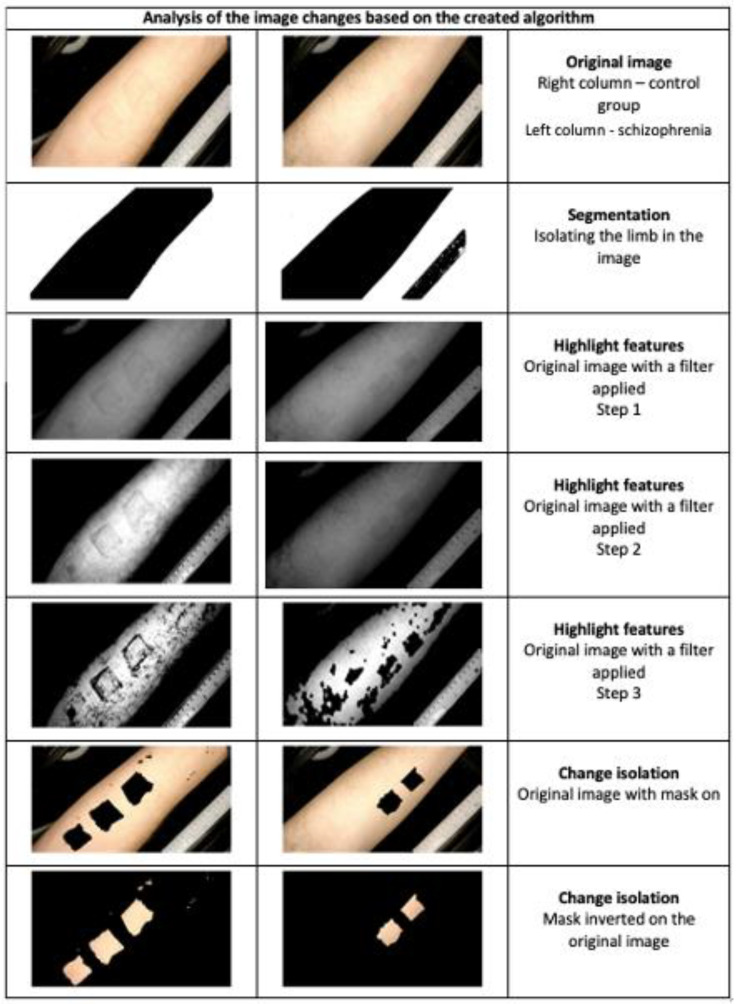
The analysis of photos was subjected to the created algorithm to isolate the changes occurring on the skin during NSFT using the SKINREMS device [33].

In Figure 3 we present an example of a trial conducted on a patient suffering from SCH and a person from the control group.

We believe that the new method of NSFT assessment may be a simple tool to enable accurate differentiation of patients with SCH and BD and the identification of the patients group with observed disorders of membrane lipid metabolism, who should be expected to have a favorable therapeutic response to the implementation of unsaturated fatty acid supplementation and limit the amount of saturated and trans-unsaturated fatty acids in the diet.

In our previous studies, without the use of the designed measuring system, the sensitivity and specificity of the performed NSFT were 71% and 66%, respectively, for the compared SCH patients and the control group; 55% and 54%, respectively, for the compared BD and the control group; and 91% and 72%, respectively, for the compared SCH and the BD patients [33]. It is expected that the usage of a newly designed measuring device will increase both test parameters.

#### 3.1.3. Diagnostic Accuracy of the NSFT

NSFT has been performed over the years involving patients suffering from schizophrenia and affective disorders. Table 1 is based and modified on Sun et al. and presents the diagnostic accuracy of NSFT, arranged from the highest sensitivity method used [43].

#### 3.1.4. Applicability of NSFT for High-Risk Psychosis

Clinically defined help-seeking UHR individuals seem to require intensive monitoring. Noting the fact that psychotic outbreaks often occur in the most productive age of early adulthood, it is crucial to ensure early intervention [48]. Diverse clinical approaches attempt to prevent full-blown psychotic disorders based on available criteria and concepts of prodromal symptoms [49,50]. As the use of antipsychotic drugs in the prevention of psychosis is controversial, there are studies showing the validity of PUFA supplementation [51].

The role of emerging studies that present different dynamics of NSFT reactions depending on the progression of the disease should be emphasized [52,53]. Langbein et al. showed various reactions to niacin solution across different UHR groups (UHR-B—BLIPS group, UHR-A—attenuated symptoms group, and UHR-G—genetic risk group) based on PACE criteria [54].

Moreover, Smesny et al. maintain that PUFA supplementation leads to the normalization of PLA2 activity, implying protective properties against the onset of a psychotic episode [55].

#### 3.1.5. Factors That Can Affect the NSFT Results

##### Sociodemographic Characteristics

The studies of Nilsson et al. showed lower electrodermal activity after auditory stimulation in patients with SCH after orally taking niacin. However, neither ectodermal activity nor niacin sensitivity correlated with their age [21]. Taking into consideration the double hit hypothesis, other reports by Nilsson et al. support the assumption of neurodevelopmental disturbance affecting higher cortical and autonomic function, which reflected the niacin test results. Still, in this case, niacin non-responders did not differ in age from responders [30]. Yao et al., examining the skin reaction with a laser doppler flowmeter in individuals diagnosed with SCH and BD, also proved that niacin response abnormality was not influenced by the age or race of the patients [41].

Despite studies that do not confirm age as a confounder, some findings challenge this position [18,23,56]. Smesny et al. showed the influence of age and the differences in the response of men and women on the NSFT results [57]. Generally, women showed a stronger skin reaction to the NSFT, but the reaction became weaker with the increasing age of the women. Not only the widely discussed protective effect of estrogens on the onset of SCH, but also the menstrual cycle, could be important in deliberating skin re-actions in the NSFT [58,59,60]. In another study, Wang et al. showed significant effects in the case of gender, alcohol drinking, and education, but only at specific niacin concentrations [45]. On the other hand, Nilsson et al. did not show a difference in education years between non-responders and responders [30].

##### Using Psychoactive Substances

Based on clinical and theoretical considerations by David Horrobin, that prostaglandin deficiency is associated with patients diagnosed with SCH as well as those abusing alcohol, it is worth noting that the results of the Fiedler et al. study showed impaired skin reaction in NSFT in individuals addicted to alcohol [35,61,62]. Taking substance use into consideration, there are reports confirming that smoking cigarettes do not affect the NSFT results [41,46]. Likewise, Smesny et al. presented the results of the study that showed no observable effect on skin redness during the NSFT in cannabis users [42]. In another publication by Smesny et al., cannabis smoking also did not influence niacin flushing. Nevertheless, observations showed that cannabis use was much more common in first-episode compared to multi-episode patients [63]. However, there are also research results that show the influence of smoking cannabis on healthy individuals’ skin reactions. In such subjects, the redness of the skin during NSFT was less intense [64]. Additionally, the study of Chang et al. showed that the consumption of coffee did not affect the NSFT results. Although, some studies showed that only the age difference between relative and proband and the coffee drinking status affect the flush response [39]. The history of allergy in the family was also irrelevant [65].

##### Factors Related to the Course and Treatment of Psychosis

As for the duration and medication of the illness, in a study by Nilsson et al., these factors do not correlate with niacin sensitivity and have no significant effect on the NSFT results [21,30]. In a study conducted by Liu et al., no niacin concentration showed significance on skin flushing regarding a medication with neuroleptics or mood stabilizers [46]. Similarly, in a study by Bosveld-van Haandel et al., antipsychotics did not appear as confounders [25]. There are studies claiming that the number of hospital admissions did not matter in response to niacin flushing [64]. Based on a study by Rybakowski et al., even a family history of SCH has no bearing on the test result [23].

##### The Intensity of Psychopathological Symptoms and Difficulties in Everyday Functioning

Both the studies by Nilsson et al. and Liu et al. showed that the Positive and Negative Syndrome Scale (PANSS) had a nonsignificant effect on the NSFT results [21,30,46]. Taking into account Global Assessment Functioning (GAF), in the study by Yao et al., no significant correlations were found between the scores of the scale and NSFT outcomes [41].

In contrast, 1990 Revised Symptom Check List (SCL 90-R) showed an association between reduced skin flushing and high SCL 90-R ratings [63]. In a study by Glen et al., it turned out that on the Montgomery Asberg Depression Rating Scale (MADRS) and the Nurses’ Observation Scale for Inpatient Evaluation (NOSIE), flushers scored significantly higher than subjects whose reaction to niacin was disturbed [34]. Interestingly, by using the Brief Psychiatric Rating Scale (BPRS), Scale of Assessment of Positive Symptoms (SAPS), and Scale of Assessment of Negative Symptoms (SANS), Smesny et al. obtained results indicating no significant difference between first-episode and multi-episode patients. Moreover, neither total scores nor individual subscales correlated significantly with response to the NSFT [63].

##### Genetic Pathways and the Response to NSFT

Concerning pathogenesis at the molecular level, no association has yet been found between phospholipid abnormalities and an impaired response to the NSFT. There were attempts to assess mRNA levels of genes of the PLA2/COX cascade, which have shown some regularities. Yang et al. showed that five single nucleotide polymorphisms in PTGS2 and one PLA2G4A were significantly associated with the degree of response to NSFT, thus causing the disturbance in the free AA catabolism and inducing overexpression of IL-6. In the study, the level of CREB1, COX-2, and the PGE2 receptor EP4 were downregulated, which may be a significant trace to a poor reaction in the niacin test [66].

Covault et al. showed that C to T single nucleotide polymorphism in the first intron of the FACL4 gene for long-chain fatty acid-CoA ligase type 4 involved in AA, EPA, and DHA metabolism is associated with a stronger skin reaction in the niacin test in SCH patients and in the control group. In addition, a significant excess of the T allele was observed in individuals suffering from major depression compared with controls (49% vs. 38%; *p* = 0.003) and a non-significant excess of the T allele was observed in SCH patients (44%; *p* = 0.29). Moreover, male SCH patients with the T0 genotype showed similar erythema to males from the control group with C0 genotype but reduced when compared with the control group with T0 genotype [67].

A study by Chang et al. that compared the skin reaction to the niacin flush test in families with only one person diagnosed with schizophrenia and those with a pair of affected siblings showed more impaired niacin flushing in SCH patients and relatives from families with higher genetic loading [65].

A study by Nadalin et al. focused on the etiology of poor response to the NSFT in patients with SCH based on the two functional A/G polymorphisms of the PLA2G4A and PTGS2 genes and the content of fatty acids in red blood cells. Both polymorphisms had a statistically significant impact on the NSFT results showing G alleles responsible for more intense niacin reactions. Additionally, PUFA values were significantly reduced in patients. However, their association with niacin sensitivity was not detected by the test methodology used by the researchers [68].

**Figure 4:**
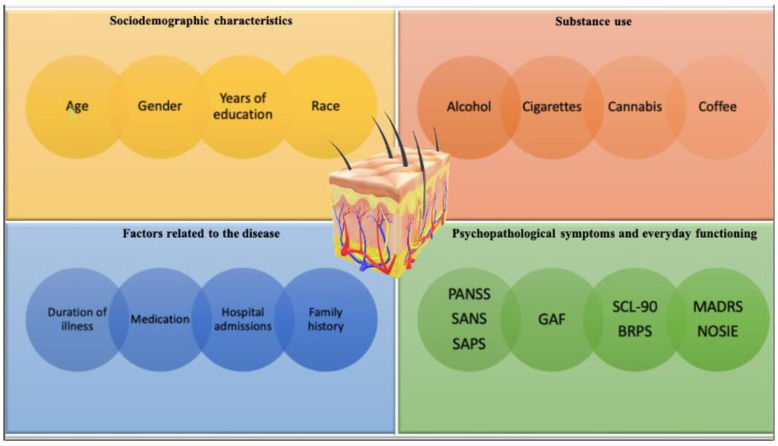
Studied factors that could potentially affect the results of the NSFT. *based on the articles discussed above: PANSS—The Positive and Negative Syndrome Scale, SANS—Scale for the Assessment of Negative Symptoms, SAPS—Scale for the Assessment of Positive Symptoms, GAF—Global Assessment of Functioning Scale, SCL-90—The Symptom Checklist-90, BRPS—Brief Psychiatric Rating Scale, MARS—Montgomery-Asberg Depression Rating Scale, NOSIE—Nurses’ Observation Scale for Inpatient Evaluation.

Furthermore, Nadalin et al. tested polymorphic variants for the PLA2G6 and PLA2G4C genes, which encode calcium-independent phospholipase A2 beta (iPLA2β) and cytosolic phospholipase A2 gamma (cPLA2γ) enzymes that mediate phospholipid remodeling and replenish the AA reservoir for prostaglandin synthesis. Nevertheless, this study did not demonstrate an influence of genetic polymorphisms on the NSFT results [69]. Studied factors that could potentially affect the results of the NSFT are presented in Figure 4. 

### 3.2. From Horrobin’s Theory to the Future

Adaptation of staging in psychiatry raises hopes for the treatment of the disease’s initial phase, which implies greater efficiency, safety, and precision and entails lower economic, social, and emotional burdens for the patient [70]. As the first episode of SCH appears most frequently in late adolescence or early adulthood, and prodromal symptoms occur from 3 to 5 years before the full outbreak of the disease, precise staging seems to be the key to the prevention and early intervention of vulnerable individuals [71].

Over the years, concepts for the course of SCH have been put forward, beginning with Fava and Kellner in 1993, then the Mark Agius model, which then led to an even more extensive concept by McFarlane et al. [72,73,74]. So far, the most comprehensive clinical staging model framework for psychotic and severe mood disorders was developed by McGorry et al. The model specifies the characteristics of the disorder clinical picture, a proposal for an appropriate intervention at a given stage, and distinguishes specific biological markers. Interestingly, in the early stages, it proposes a simple, non-invasive NSFT as a potential test to estimate the risk of psychosis [75]. At this point, returning to the assumptions of David Horrobin, who called SCH a prostaglandin deficiency disease, one should focus on the significance of AA, which forms the basis of the skin reaction in the NSFT [35].

Since Horrobin suggested the NSFT as a screening for SCH, there have been attempts to adapt the test to artificial intelligence technology that could be widely available. Such activities create the concept of individualized medicine and allow for a holistic approach to the patient’s difficulties, giving hope for simple solutions such as modification of the diet and lifestyle [76].

### 3.3. The Hope Hidden in Diet

It has long been known that dietary interventions have the potential to improve both the physical and mental health of an individual. Unfortunately, prioritizing such recommendations in daily practice is extremely rare, and nutritional psychiatry is just beginning to show how food choices can affect SCH spectrum disorders [77]. Even if there are currently limited nutritional guidelines for mental health issues, the World Federation of Societies of Biological Psychiatry (WFSBP) and the Canadian Network for Mood and Anxiety Disorders (CANMAT) contributed to the creation of clinician guidelines for the treatment of psychiatric disorders with nutraceuticals and phytoceuticals. They emphasize the preventive role of omega-3 fatty acids in the transition to psychosis in high-risk youth with pre-existing fatty acid deficiency [78]. As polyunsaturated fatty acids (PUFAs) are the major constituents of neuronal membranes, their meaning in mental disorders is crucial [79].

**Figure 5:**
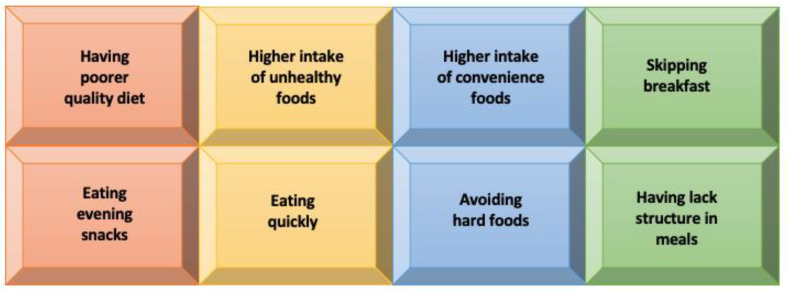
Eating habits in patients who have experienced psychosis.

Almost 100 years ago, Burr and Burr discovered the importance of linoleic (LA) and alpha-linolenic acid (ALA) and created the term “essential fatty acids”, since they must be supplied to the organism along with the diet [80]. A substantial point of PUFA metabolism is that n-3 and n-6 PUFAs compete for the same delta-6-desaturase enzyme. Hence, the basis of their optimal transformation is the proportion in which they are delivered [81]. Industrialization resulting in dynamic changes in diet is undoubtedly a new phenomenon in the history of mankind. It has led, among other things, to an increase in the consumption of saturated fat, omega-6 fatty acids, and trans-fatty acids, decreasing omega-3 fatty acids intake. Additionally, trans-fatty acids interferes with the desaturation and elongation of omega-6 and omega-3 acids, which leads to a reduction in the availability of AA in human metabolism [82,83]. The ratio of omega-6 and omega-3 fatty acids in a Western diet, poor in omega-3, fluctuates within 15–20: 1 instead of 1:1 to 4:1 [84,85]. It has been proven that a significantly increased omega-6 to omega-3 ratio is associated with the occurrence of various chronic diseases including inflammatory bowel disease, cardiovascular disease, rheumatoid arthritis, and mental disorders [86]. Additionally, in the aspect of eating habits, there are systematic review reports on the dietary patterns of individuals suffering from psychotic disorders that are worth paying attention to [87]. Based on discussed articles some eating habits of patients are presented in Figure 5.

There is much promising evidence for the pivotal meaning of PUFA in terms of dietary research on mental disorders. Robinson et al. report that omega-3 supplementation has the potential to relieve depression and anxiety symptoms in subjects who have recently experienced psychosis [88]. Jones et al. also reported protective effects of long-chain omega-3 and omega-6 fatty acids in SCH. It has been suggested, however, that individuals suffering from SCH may have a disturbed mechanism for converting short-chain to long-chain PUFAs [89]. Moreover, some speculate that PUFA biomarkers may be useful in identifying individuals with deteriorating neurocognitive functioning, which entails a worse prognosis. The basis for this statement is the results of the study conducted by McLaverty et al., who emphasize the importance of PUFAs, especially in terms of verbal fluency in the UHR population [90]. Interesting results regarding PUFA supplementation are presented by Hsu et al. who claim that supplementation with omega-3 acids reduces the conversion rate to psychosis and has a positive effect on both positive and negative symptoms as well as global functioning in the case of UHR adolescents [91].

Moreover, it appears that omega-3 fatty acids have a biological effect similar to antipsychotic drugs, with the advantage that they have no side effects and favorably affect the metabolic state of a patient. Otherwise, they prove to be effective even before the first symptoms of SCH appear, where antipsychotics are not applicable. Notwithstanding the low cost of supplementation has its strengths, but it is possible that in terms of personalized medicine, only selected subjects will be able to benefit from it [92]. Some authors report that omega-3 supplementation is most effective in patients in the prodromal phase or early onset stages, which, concerning niacin screening trials, gives hope for faster treatment [93]. There is also evidence that the prompt identification of patients with omega-3 fatty acid shortage is essential to obtain the maximum benefit from such an intervention [94]. Nevertheless, in opposition to these observations, some studies report that decreased levels of essential fatty acids among patients with schizophrenia may be related not to the disease itself but to other independent factors, such as cigarette smoking status [95].

## 4. Conclusions

There is an urgent need to improve NSFT as a simple, non-invasive, reproducible, cheap method that can be of potential importance in the following areas:

1. Creating a new classification and diagnosis of mental disorders, especially psychosis, based on pathophysiological premises.
2. Searching for new therapeutic options and drugs based on the mechanisms of NSFT action.
3. Early intervention and staging in psychiatry.
4. Defining an individualized diet for patients, which may be a factor in alleviating symptoms and perhaps even leading to remission and its maintenance.

Considering the above, there is an opportunity to improve diagnostics, which can contribute to reducing the individual, social, and economic burden of a debilitating disease, and starting treatment on time would prevent its unnoticed, insidious, and destructive course. More scholars should consider studies investigating the niacin skin flush test outcomes connected with physiological indicators such as EEG and near-infrared techniques as well as various biological markers. The specificity of NSFT should be explored from multiple dimensions to provide more conclusive evidence of its clinical application.

## Figures and Tables

**Table 1:** Diagnostic accuracy of NSFT in individual studies.

	Study	Year of Publication	Individuals	Country	Sensitivity	Specificity	Type of Data
1.	Smesny et al. [42]	2003	25 SCH	Australia	92.0%	84% in SCH and HCs	Quantitative
			25 HCs				
2.	Karakula-Juchnowicz et al. [33]	2020	56 SCH	Poland	91%	72% in SCH and BD	Semi-quantitative
			29 BD		71%	66% in SCH and HCs	
			45 HCs		55%	54% in BD and HCs	
3.	Puri et al. [19]	2001	21 SCH	UK	90%	75% in SCH and HCs	Semi-quantitative
			20 HCs				
4.	Smesny et al. [42]	2003	25 SCH	Australia	84.0%,	76% in SCH and HCs	Semi-quantitative
			25 HCs				
5.	Ward et al. [18]	1998	35 SCH	UK	83%	77% in SCH and HCs	Semi-quantitative
			22 HCs				
6.	Horrobin [37]	1980	N/A	UK	80%	N/A	Qualitative
7.	Puri et al. [38]	2002	27 SCH	UK	77.8%	65.38% in SCH and HCs	Semi-quantitative
			26 HCs				
8.	Messamore et al. [20]	2003	27 SCH	USA	74.0%	81% in SCH and HCs	Quantitative
			21 HCs				
9.	Ross et al. [40]	2004	27 SCH	Canada	70.0%	86% in SCH and HCs	Quantitative
			26 BD			81% in SCH and BP	
10.	Hu et al. [44]	2022	82 SCH	China	57%	89%	Quantitative
			41 BD				
			80 HCs				
11.	Wang et al. [45]	2021	307 SCH	China	55.28%	83.56% in SCH or AD and HCs	Semi-quantitative
			179 BD				
			127 UD				
			148 HCs				
12.	Glen et al. [34]	1996	126 SCH	UK	52%	N/A	Qualitative
13.	Liu et al. [46]	2007	61 SCH	Taiwan	49.2%,	92.5% in SCH and HCs	Semi-quantitative
			18 BD			88.2% in SCH and BP	
14.	Hudson et al. [47]	1999	23 SCH	Canada	43.0%	97% in SCH and HCs	Quantitative
			30 HCs				
15.	Hudson et. al. [22]	1997	33 SCH	Canada	42.9%,	94.4% in SCH and BP	Quantitative
			18 BD			100% in SCH and HCs	
			28 HCs			97.8% in SCH, BP, and HCs	
16.	Sun et al. [43]	2017	163 SCH	China	23.3–42.0%	82.4%-88.9%	Semi-quantitative
			63 MDD				
			63 HCs				
17.	Yao et al. [41]	2015	70 SCH	USA	30.0%	95% in SCH and HCs	Quantitative
			59 BD			97% in SCH and BP	
			90 SCH		32.0%	95% in SCH and HCs	
			93 HCs			87% in SCH and MDD	
18.	Rybakowski et al. [23]	1991	33 SCH	Poland	24%,	100% in SCH and AD	Qualitative
			18 AD				
19.	Tavares et al. [27]	2003	38 SCH	Brazil	23.7%	85.8% in SCH and HCs	Semi-quantitative
			28 HCs				
20.	Lin et al. [39]	2006	153 SCH	Taiwan	13.7%	96.8% in SCH and HCs	Semi-quantitative
			94 HCs				

SZ: schizophrenia, BD: bipolar disorder, HCs: healthy controls, UD: unipolar depression, MDD: major depressive disorder, AD: affective disorder.

## Data Availability

The data presented in this study are available on request from the corresponding author.

## Abbreviations

NSFT—niacin skin flush test, SCH—schizophrenia, SCHAD—schizoaffective disorders, BD—bipolar disorder, B-SNIP—Bipolar-Schizophrenia Network for Intermediate Phenotypes, UHR—ultra-high risk, DSM-5—Diagnostic and Statistical Manual of Mental Disorders, AA—arachidonic acid, PLA2—phospholipase A2, PANSS—Positive and Negative Syndrome Scale, GAF—Global Assessment Functioning, SCL 90-R—Symptom Check List 1990 Revised, MADRS—Montgomery Asberg Depression Rating Scale, NOSIE—and Nurse’s Observation Scale for Inpatient Evaluation, BPRS—Brief Psychiatric Rating Scale, SAPS—Scale of Assessment of Positive Symptoms, SANS—Scale of Assessment of Negative Symptoms, PUFAs—polyunsaturated fatty acids, LA—linoleic acid, ALA—alpha-linolenic acid, WFSBP -World Federation of Societies of Biological Psychiatry, CANMAT—Canadian Network for Mood and Anxiety Disorders, COX—cyclooxygenase, PGG_2_—prostaglandin G_2_, PGH_2_—prostaglandin H_2_, PGD_2_—prostaglandin D_2_, PGE_2_—prostaglandin E_2_
